# A profile of research on the parasitic trypanosomatids and the diseases they cause

**DOI:** 10.1371/journal.pntd.0010040

**Published:** 2022-01-13

**Authors:** David Horn

**Affiliations:** The Wellcome Trust Centre for Anti-Infectives Research, Division of Biological Chemistry & Drug Discovery, School of Life Sciences, University of Dundee, Dundee, United Kingdom; Instituto de Investigaciones Biotecnológicas, ARGENTINA

## Abstract

The parasitic trypanosomatids cause lethal and debilitating diseases, the leishmaniases, Chagas disease, and the African trypanosomiases, with major impacts on human and animal health. Sustained research has borne fruit by assisting efforts to reduce the burden of disease and by improving our understanding of fundamental molecular and cell biology. But where has the research primarily been conducted, and which research areas have received the most attention? These questions are addressed below using publication and citation data from the past few decades.

## Introduction

The parasitic trypanosomatids include *Leishmania* spp. and the African and South American trypanosomes. The most serious form of leishmaniasis [1] is visceral leishmaniasis, or kala-azar, which is typically fatal without treatment. There are estimated to be approximately 70,000 new cases each year. Post-kala-azar dermal leishmaniasis (PKDL) can also occur after apparent cure. The most common form of leishmaniasis is cutaneous leishmaniasis, which causes skin lesions and ulcers and lifelong scarring; there are estimated to be approximately 800,000 new cases each year. An even more destructive form is mucocutaneous leishmaniasis. The *Leishmania* parasites are transmitted by female phlebotomine sandflies.

Chagas disease, or American trypanosomiasis [2], is caused by *Trypanosoma cruzi*. More than 6 million people are thought to be infected worldwide, and up to 30% are expected to develop cardiac, digestive, or neurological, and potentially life-threatening symptoms, often many years after the initial infection. American trypanosomes are transmitted by triatomine bugs; other important transmission routes are oral, congenital, via blood transfusion, or via organ transplantation.

Sleeping sickness, or human African trypanosomiasis [3], which is often fatal without treatment, is caused by *Trypanosoma brucei gambiense* (west and central Africa) or *Trypanosoma brucei rhodesiense* (east and southern Africa), with *T*. *b*. *gambiense* accounting for >98% of current cases. The latest major sleeping sickness epidemic started in the late 1970s, peaked in the late 1990s, and continued into the 21st century. The disease remains endemic in several sub-Saharan African countries but with <1,000 cases reported in 2018. Animal African trypanosomiasis is caused by *Trypanosoma congolense*, *Trypanosoma vivax*, or *T*. *b*. *brucei* in wild and domestic animals; the disease is known as nagana in cattle. The African trypanosomes are typically transmitted by tsetse flies (*Glossina*).

Leishmaniasis, Chagas disease, and human African trypanosomiasis (who.int/news-room/fact-sheets) are among 20 neglected tropical diseases (NTDs) currently within the World Health Organisation (WHO) portfolio (who.int/neglected_diseases/diseases/). All 3 diseases have been targeted for elimination as part of the WHO road map for NTDs 2021–2030 (WHO/UCN/NTD/2020.01).

My hope is that a broad overview of research on the trypanosomatids and the diseases they cause will be of potential interest to students, researchers, and many others interested in research on NTDs. Below, I use publication and citation data from the past few decades to explore where research in these areas has been primarily conducted, and which research areas have received the most attention over time.

## Methods

Data relating to publication outputs were downloaded from PubMed (pubmed.ncbi.nlm.nih.gov) on 24:09:2021, using the terms “leishmania,” “cruzi,” “brucei,” “congolense,” or “vivax” (AND “trypanosoma” in the last 2 cases). Data relating to citations were downloaded from Scopus (http://www.scopus.com) on 10:10:2021, using the terms above and also “trypanosome(s),” “leishmaniasis,” “Chagas,” “sleeping,” AND “sickness.” The top 5% most highly cited (HC) articles from each year (cites per year since publication) were extracted, and duplicates, reviews, and articles with a focus on neither human nor veterinary parasitic trypanosomatids were removed, yielding the “highly cited” set of 1,786 articles, average of 58 from each year with a range from 35 (1991) to 88 (2018). Each article was assigned to a primary trypanosomatid and to one from a set of primary research area or topic terms; the latter was achieved for >90% of articles. All topics with >9 HC articles assigned are detailed in the figures. The titles from the “‘highly cited” articles, minus the terms used to search Scopus, were used to derive a word cloud for each trypanosomatid group (https://www.wordclouds.com/).

Articles cited here include a set of 3 seminar-style reviews recently published in the *Lancet* (*n =* 3), one for each major disease area; all research articles published since 1990 that had previously been cited >20 times per year since publication (www.scopus.com; *n* = 88) and an additional 16 articles cited >17 times per year since publication. These articles cover a wide range of research areas and topics and serve as exemplars for those research areas. Further citations include a set of research articles that appeared to be “ahead of their time,” preceding other HC articles on the same topic by >5 years (*n =* 3); a set of research articles from 2020 or 2021 (*n* = 6), since recent articles are underrepresented in the HC set; and a set of research articles describing further novel features first discovered in trypanosomatids (*n* = 7). The 5 key papers were selected to reflect impacts in the areas of immunity, host–parasite interactions, omics, drug discovery, and the delivery of new therapies.

### Publication outputs by country

**Fig 1:**
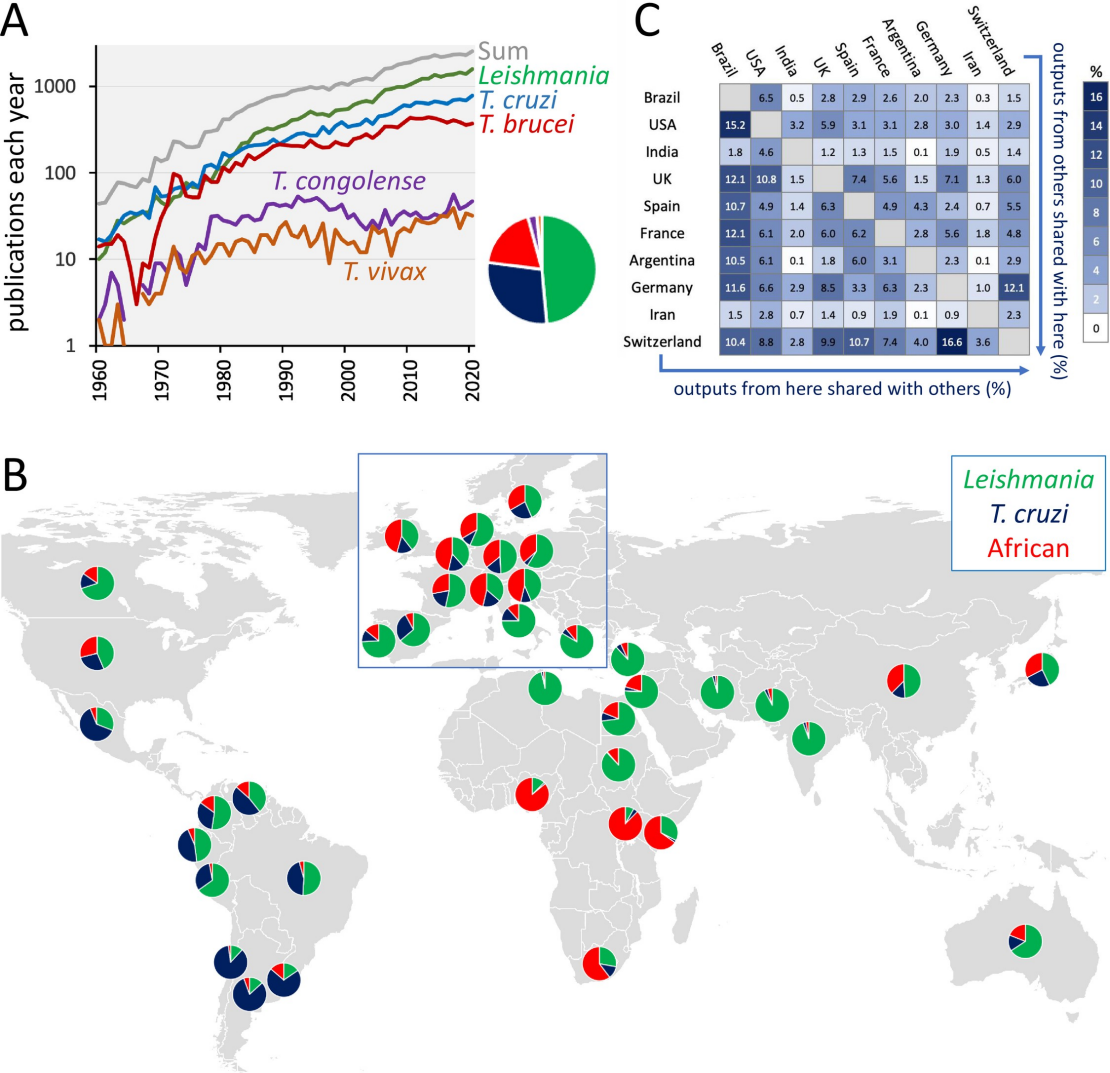
Global trypanosomatid research outputs. (**A**) Global trypanosomatid publication outputs in PubMed from 1960–2020; 58,784 outputs in total. The pie chart indicates the distribution of outputs among the trypanosomatids. (**B**) Trypanosomatid publication output affiliations in PubMed from 1995 to 2020 for 39 countries; the African trypanosomes, “brucei,” “congolense,” and “vivax” are combined here. The World map was obtained from Wikimedia Commons (https://commons.wikimedia.org/wiki/File:BlankMap-World-Flattened.svg). (**C**) International collaboration is illustrated on a heat map indicating pairwise joint affiliation, according to PubMed, over the past 10 years, and for those 10 countries listed on the most outputs.

The first graph (Fig 1A) shows global life sciences and biomedical publication outputs including the terms “leishmania,” “cruzi,” “brucei,” “congolense,” or “vivax” (and “trypanosoma” in the last 2 cases) in PubMed and published in the 61-year period from 1960 to 2020. There were 31,281 “leishmania,” 18,336 “cruzi,” 12,088 “brucei,” 1,684 “congolense,” and 1,000 “vivax” publications; 58,784 in total, 40% of which were published in the last 10 years. For comparison, there have been a similar number of “plasmodium” (malaria) publications in this time frame (approximately 60,000), also with a similar proportion published in the last 10 years. The figure for “cancer” is 3.4 million. The overall rate of increase in trypanosomatid outputs was accelerated between 2005 to 2010 and has averaged more than 6 publications a day for the past 5 years. All groups, except for *T*. *brucei*, display a continuing steady increase in outputs over the past 10 years. Fig 1B shows outputs for the 3 main parasite groups since 1995 and for 39 countries on a global map. The pattern that emerges is largely explained by disease endemicity but also reflects international connectivity, which in some cases has its historical roots in colonialism. To further illustrate connectivity, proportions of international collaborative publications are illustrated on a heat map (Fig 1C). As examples, the UK appears on 2.8% of publications listing Brazil as an affiliation, while Brazil appears on 12.1% of publications listing the UK; India appears on 3.2% of publications listing the USA as an affiliation, while the USA appears on 4.6% of publications listing India. Higher degrees of international collaboration reflect proximity in terms of geography, and shared topic focus, while funding structures may also have an impact here.

**Fig 2:**
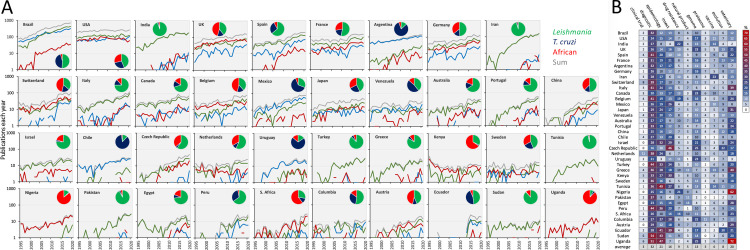
Trypanosomatid research output trends by country. (**A**) Trypanosomatid publication output trends in PubMed from 1995 to 2020 for 39 countries. Countries are arranged in order of total number of “trypanosomatid” outputs; from 8,799 to 147. The African trypanosomes, “brucei,” “congolense,” and “vivax” are combined here. The pie charts indicate the distribution of outputs among the trypanosomatids. (**B**) The heat map indicates proportions of outputs from each country associated with each of the topics listed, according to PubMed over the past 10 years.

Fig 2 shows outputs for the 3 parasite groups for the countries detailed above on a timeline between 1995 to 2020. Before 2012, only 2 countries (Brazil and USA) produced >100 “trypanosomatid” outputs in consecutive years. Seven additional countries (UK, India, Spain, France, Argentina, Germany, and Iran) have now sustained this level of output for several consecutive years. Brazil, India, and Iran, in particular, display a remarkable increase in “leishmania” outputs, accounting for 7% of global output from 1990 to 1994 and 48% from 2016 to 2020.

The terms “visceral” or “cutaneous” (leishmaniasis) occur at a similar level in outputs from Brazil (33% versus 32%), while “visceral” is overrepresented in outputs from India (57% versus 10%) and “cutaneous” is overrepresented in outputs from Iran (53% versus 23%). Countries that have sustained a higher level of “leishmania” outputs than for the other trypanosomatids include the USA, India, Spain, France, Germany, Iran, Italy, Canada, Australia, Portugal, Israel, Turkey, Greece, Tunisia, Pakistan, Egypt, and Sudan. Countries that have sustained a level of “cruzi” outputs that is higher than for the other trypanosomatids include Argentina, Mexico, Venezuela, Chile, and Uruguay. Countries that have sustained a level of African trypanosome outputs that is higher than for the other trypanosomatids for at least half of the time frame analysed include the UK, Switzerland, Belgium, Kenya, Nigeria, South Africa, and Uganda.

Data on topic focus by country over the past 10 years are illustrated using a heat map (Fig 2B). Notable features include particularly high relative levels of activity in insect vector research and evolutionary biology in the Czech Republic and Ecuador; in drug resistance in India, Belgium, Nigeria, and South Africa; in vaccine research in Iran; and in veterinary research in Italy, Greece, Nigeria, and Uganda. There has been a high relative level of activity in diagnostics in all countries listed.

### Publication outputs by topic

Details for journal articles in the fields of life science and health science and published in the 32-year period from 1990 to 2021 were identified in Scopus using a set of trypanosomatid and trypanosomal disease terms, yielding >50,000 outputs (see Methods). To capture those research topics that have received the most attention, the 5% most highly cited (HC) articles from each year (to 2020) were identified and assigned to a primary trypanosomatid group, yielding 1,021 HC articles for “leishmania” (57%), 398 for “cruzi” (22%), and 367 for the African trypanosomes (21%), 1,786 HC articles in total. A notable feature within this set of articles was that median author number increased from 4 between 1990 to 1995 to 9 between 2015 to 2020. Prominent research topics captured within this set are detailed below, where citations have been selected based on citation counts (see Methods).

### Trials, diagnostics, surveillance, and vectors

**Fig 3:**
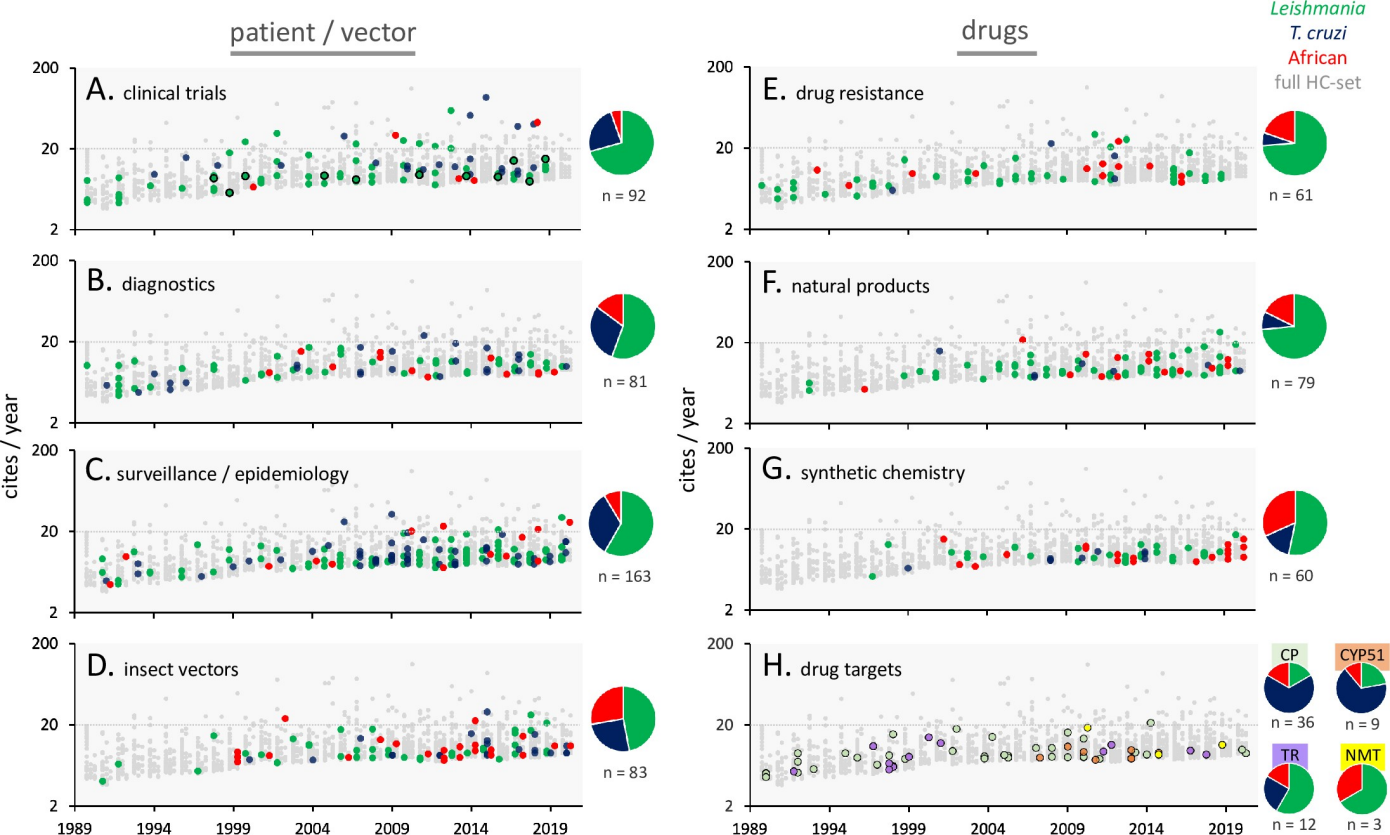
Citation rates by trypanosomatid research topic. HC articles from 1990–2020 are highlighted for each of the patient/vector (**A-D**) and drug-related topics (**E-H**), using data from Scopus. The grey data points indicate the full HC set of 1,786 articles, with “leishmania” articles displaced to the left and “African trypanosome” articles displaced to the right in each year-group. In (A), vaccine trials are indicated by symbols with a black outline. For drug targets in (H), each target is indicated using different coloured symbols; CP, CYP51, NMT, TR. The pie charts indicate the distribution of outputs in each category among the trypanosomatids. CP, cysteine peptidase; CYP51, sterol-14α-demethylase; HC, highly cited; NMT, *N*-myristoyltransferase; TR, trypanothione reductase.

Among articles reporting the results of clinical trials (92 HC articles; Fig 3A), several have been particularly HC in recent years (2013 to 2018). Reports describe primarily drug trials, which, against the leishmaniases, include assessments of antimony [4], miltefosine [5,6], liposomal amphotericin B (AmBisome) [7], paromomycin [8,9], or combination therapies [10], as well as thermotherapy to treat cutaneous leishmaniasis. Trials against Chagas disease include assessments of benznidazole alone [11,12] or in combination with posaconazole [13,14] or the ravuconazole prodrug E1224 [15], or nifurtimox alone. Trials against the advanced CNS stage of sleeping sickness include assessments of melarsoprol, a nifurtimox–eflornithine combination [16], or fexinidazole [17]. Safer therapies and new dosing regimens have been introduced as a result, reducing adverse events, and improving efficacy. Disappointingly, benznidazole failed to reduce cardiac clinical deterioration in Chagas patients in the BENEFIT trial [11], and both posaconazole [13,14] and E1224 [15] failed to outperform benznidazole against chronic (CHAGASAZOL trial) or asymptomatic Chagas (STOP-CHAGAS trial), possibly due to suboptimal dosing. New oral therapies, however, such as miltefosine for leishmaniasis [5] and fexinidazole for sleeping sickness [17], were shown to be safe and effective, promising to facilitate administration and to address issues associated with compliance. The recently reported BENDITA trial suggested that shorter and reduced benznidazole regimens could be effective against Chagas disease [18], and finally, the boron-containing drug, SCYX-7158 [19], now known as acoziborole, is showing great promise in ongoing trials as an oral therapy for sleeping sickness.

Although no vaccines have been approved for wider use, there have been several antileishmanial vaccine trials in humans, to assess safety using a recombinant adenoviral vaccine, or efficacy using either whole autoclave-killed *Leishmania* or recombinant antigens, typically yielding moderate degrees of efficacy. Several antileishmanial vaccine trials in dogs, using either recombinant antigens or antigens secreted from *Leishmania infantum*, show good efficacy, suggesting a potential route to reducing the parasite burden in these reservoir hosts. Other trials involved the application of human host-directed immunomodulators [20] or the use of insecticide-impregnated dog collars.

Clinical studies, surveillance, and intervention are facilitated by quality diagnostic tools, and there has been substantial interest in the development and evaluation of these tools (81 HC articles; Fig 3B). PCR-based diagnostics, to detect typically high-copy number trypanosomatid sequences, have dominated [21], followed by serology, typically to detect antibodies against surface-exposed trypanosomal antigens. Xenodiagnosis also facilitates the identification of PKDL patients that continue to transmit *Leishmania* parasites to sand flies [22]. Loop-mediated isothermal amplification (LAMP) studies emerged after 2002, with LAMP primarily applied to human African trypanosomiasis. Serological tests have more recently emerged in the lateral flow based rapid diagnostic test (RDT) format. An initial positive test result may be followed up by further confirmatory tests, including enzyme-linked immunosorbent assay (ELISA), immunofluorescence antibody test (IFAT), direct detection of parasites by microscopy or, for staging human African trypanosomiasis, examination of cerebrospinal fluid for parasites, and/or elevated white blood cell count.

Surveillance and epidemiology (163 HC articles; Fig 3C) studies have focussed primarily on leishmaniasis or Chagas disease but also include efforts to map the distribution of sleeping sickness [23], to estimate populations at risk of sleeping sickness [24], and, more recently, to monitor progress in eliminating sleeping sickness [25,26]. Leishmaniasis surveillance has been largely in Iran and Brazil but also more recently in Syria, Lebanon, and Turkey. In terms of *Leishmania* reservoir hosts, many studies focus on dogs [27], with several others on cats in several European countries, and also foxes. Chagas disease surveillance has largely been in the USA [28], Brazil, and in other South American countries. Many of these studies focus on transmission via the oral or congenital route or via blood transfusion or transplantation, with additional interest in the latter routes in Europe. Cardiomyopathy has also been the subject of surveillance in chronic Chagas patients [29]. Taxonomy and typing (26 HC articles) have had an impact in the areas of diagnostics and surveillance, although genome sequencing (see below) has now largely replaced some of the earlier typing techniques. These studies provided insights into the evolution and geographic spread of *Leishmania* spp. [30] and revealed 6 discrete typing units or lineages of *T*. *cruzi*, as well as a distinct *T*. *cruzi* lineage circulating globally in bat populations.

Vector control, using long-lasting insecticides in South America and Asia, and tsetse fly traps in sub-Saharan Africa, have been effective means to control transmission of the trypanosomiases. Indeed, several surveillance studies detailed above focus on the insect vectors [31] but also consider the impact of climate change on insect range [32]. Several studies on vector biology (83 HC articles; Fig 3D) additionally deal with interactions among trypanosomatids and other insect gut–resident microbes [33], while others deal with the role of components of sand fly saliva in *Leishmania* transmission and the associated immune response. Genome sequences have been reported for trypanosomatid insect vectors [34,35] and for a tsetse endosymbiont.

### Drug research and development

The proportion of articles including the term “drug” (title, abstract, or keyword) has approximately doubled for each trypanosomatid group during the time frame analysed and has remained at approximately 40% in recent years. Sixty-one of the HC articles focus on drug resistance (Fig 3E). Miltefosine [36], amphotericin B [37], or antimonial resistance feature prominently in “leishmania” research [38]; benznidazole resistance (and nifurtimox cross-resistance) [39] in “cruzi” research; and melarsoprol–pentamidine cross-resistance [40] in “brucei” research. Defective drug uptake, particularly via aquaglyceroporins, but also increased drug efflux, feature prominently in “leishmania” and “brucei” drug resistance articles, as does metabolism of the nitro-prodrugs (13 HC articles), benznidazole, nifurtimox, and fexinidazole, by trypanosomal nitroreductase.

An increased level of interest in drug discovery emerged around 2000. This included substantial interest in natural products (79 HC articles; Fig 3F), focussing on products derived from plants [41] (including several oils), sponges, or fungi, and including frog or tarantula-derived antimicrobial peptides. Increased interest in medicinal chemistry or synthetic chemistry (60 HC articles; Fig 3G) also emerged around 2000. These studies describe the design and synthesis of a variety of compounds, sometimes inspired by natural products, and including chalcones, azoles, and quinolines. These compounds have often been tested for activity against multiple protozoa and other microbes.

Several post-2009 articles describe phenotypic drug screening (18 HC articles), using collections of compounds ranging from small sets to 1.8 million. All 3 trypanosomatid groups are represented and open-access pathogen “boxes” have emerged as a result [42]. Assessment of compounds for progression has been facilitated by screening intracellular *Leishmania* or *T*. *cruzi* parasites or by using bioluminescent imaging of parasites in mouse models [43], while drug potency assessment has been facilitated by an alamarBlue assay [44]. There have additionally been attempts to repurpose compounds approved for other clinical applications for use against trypanosomatids. Nanoparticle-based formulations for drug delivery, almost exclusively against *Leishmania* [45], also feature prominently post-2010 (44 HC articles). A notable 2002 article that describes nanoparticle formulations of the animal African trypanosomiasis drug, berenil [46], precedes other HC articles relating to nanoparticles by 7 years. Otherwise, beyond liposomal amphotericin B, these studies describe the development of gold, silver, or other lipid-based formulations against either cutaneous or visceral leishmaniasis.

Potential trypanosomal drug targets that have received substantial attention include cysteine protease [47], trypanothione reductase, sterol-14α-demethylase (CYP51), and *N*-myristoyltransferase (NMT) [48] (Fig 3H). Cysteine protease (36 HC articles), primarily from *T*. *cruzi*, has been the subject of target-based screening, following the synthesis of peptide and nonpeptide inhibitors, and virtual screening, facilitated by structural studies [49]. Proof of concept for this target has been achieved in both mouse and dog models. Trypanothione is an antioxidant and the trypanosomatid-specific analogue of glutathione. Trypanothione reductase (12 HC articles), primarily from *Leishmania*, has similarly been the subject of target-based and virtual screening, facilitated by structural studies. Development of CYP51 as a drug target (9 HC articles), primarily in *T*. *cruzi*, has also been facilitated by structural data. As stated above, however, unfortunately, the antifungal, CYP51-targeting drugs, posaconazole and E1224 [13,14], proved to be inferior to benznidazole monotherapy, and no advantages were observed in clinical trials when combining these drugs with benznidazole. Development of NMT as a target in *Leishmania* or *T*. *brucei* (3 HC articles) has been facilitated by structural data and includes characterisation of lipidated proteins and validation in mouse models of both visceral leishmaniasis and sleeping sickness.

Articles aiming to define the mechanism-of-action by which drugs kill parasites (32 HC articles) initially focused on drugs used in the clinic over many years or on natural products, several of which likely exhibit polypharmacology, yet exhibit selectivity due to specific uptake or metabolism by parasites (see above). A related set of articles focus on apoptosis-like cell death (18 HC articles) induced by these drugs and/or by oxidative stress, primarily in *Leishmania*. More recent “mechanism-of-action” studies focus on target deconvolution for new, more chemically tractable compounds, and have now revealed several new and promising specific drug targets, including the proteasome [50,51], cyclin-dependent kinase 12 [52], the kinetochore kinase CLK1 [53], and cleavage and polyadenylation specificity factor 3, the target of acoziborole [19]. Several of these new compounds are now being assessed in clinical trials.

### Host–parasite interactions

**Fig 4:**
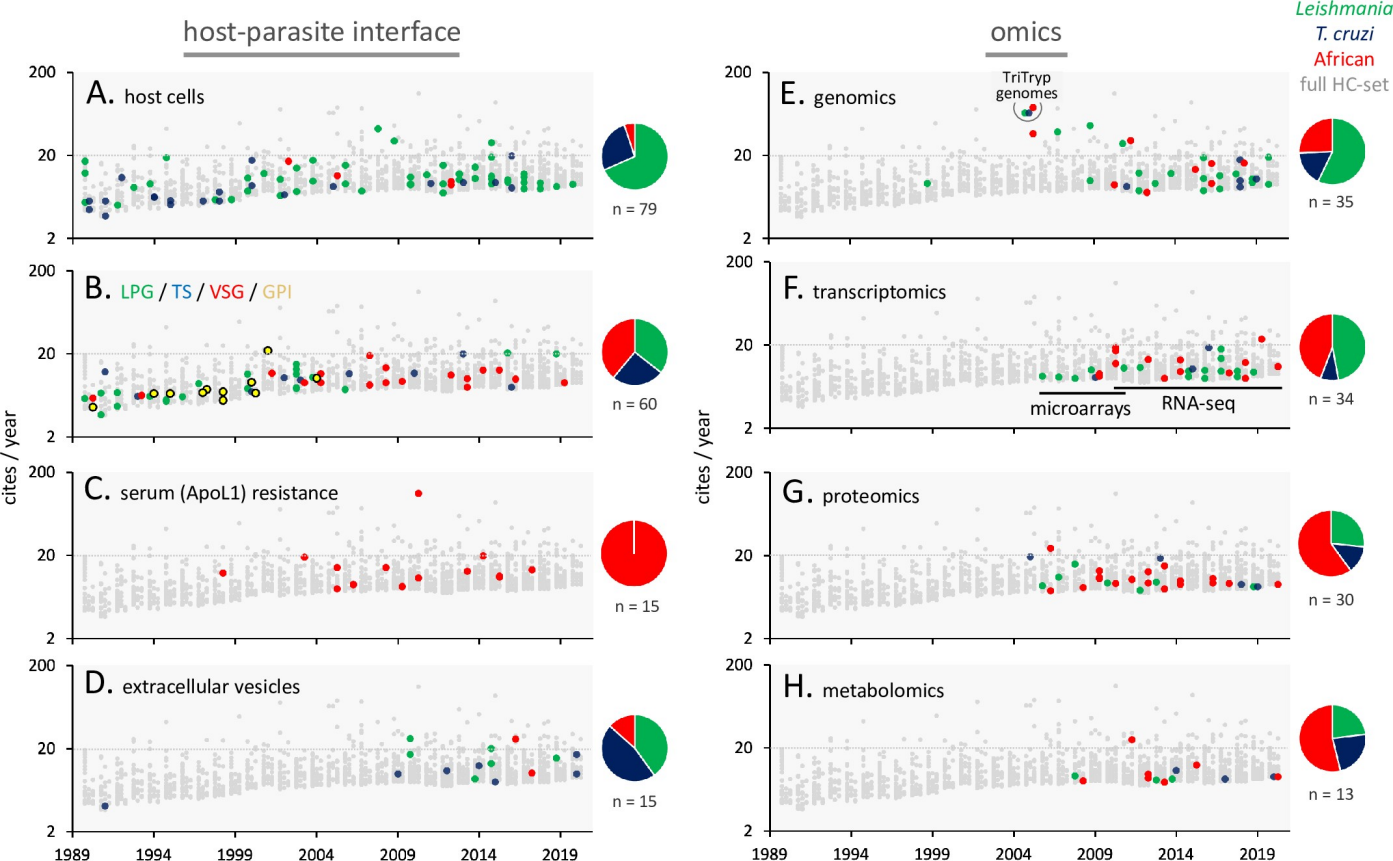
Citation rates by trypanosomatid research topic. HC articles from 1990–2020 are highlighted for each of the host–parasite interface (**A-D**) and omics-related topics (**E-H**). For parasite surface molecules in (B), each molecule is indicated using different coloured symbols; *Leishmania* LPG, *T*. *cruzi* TS, *T*. *brucei* VSG, GPI. Other details as in Fig 3. GPI, glycosylphosphatidylinositol; HC, highly cited; LPG, lipophosphoglycan; TS, *trans*-sialidase; VSG, variant surface glycoprotein.

Studies on host–parasite interactions have yielded insights into parasitic lifestyles, pathology, persistence, and life cycle progression. Host cells respond to parasites and are also modulated and exploited by parasites. Both *Leishmania* and *T*. *cruzi* parasites invade mammalian host cells and can produce dormant forms [54], presenting an additional challenge in terms of chemotherapy. Seventy-nine of the HC articles focus on host cell interactions, invasion, and modulation by trypanosomatid parasites (Fig 4A). These studies are largely based on experimental mouse models but also include studies with human cell lines and patient samples. *Leishmania* parasites replicate within parasitophorous vacuoles in host macrophages and a major theme regards establishment of infection at the sandfly bite site, which involves invasion of neutrophils [55], production of neutrophil extracellular traps (NETs) [56], and phagocytosis by macrophages; rather than controlling the infection, NETs appear to facilitate macrophage invasion. Thus, *Leishmania* parasites evade host innate immune defences and exploit host immune cells to establish infection. In contrast, the protective response at the infection site appears to depend upon monocyte-derived dendritic cells (DCs) [57].

Further studies that focus on host cell modulation by parasites also feature among this set. *Leishmania* parasites modify the parasitophorous vacuole, alter lipid and mitochondrial metabolism, and exploit host arginine by up-regulating parasite membrane transport. *Leishmania* infection induces changes in host cell DNA methylation, alters the host cell transcriptome, and represses translation, resulting in a dampened innate immune response and delayed programmed cell death. *Leishmania* RNA virus-1 can also promote parasite persistence [58]. *T*. *cruzi* parasites invade most nucleated cells via lysosomes and then escape into the cytosol, where replication depends upon host cell metabolic networks. Adipocytes appear to be important target cells, while macrophages also take up infected apoptotic cells. Several HC articles focus on the *T*. *cruzi* invasion process, involving elevation of free cytosolic calcium in the parasite, as well as recruitment of, and fusion with, lysosomes at the host cell entry site. Several additional HC articles focus on trypanosomatid calcium transporters and intracellular calcium pools, including pools within acidocalcisomes [59].

Differentiation (14 HC articles) and progression from one life cycle stage to the next also involves host–parasite interaction, with differentiation typically triggered by host-derived or other environmental signals. These studies focus on all 3 trypanosomatids, but a prominent theme relates to quorum sensing in African trypanosomes, whereby secreted parasite oligopeptidases produce an oligopeptide signal that promotes differentiation to a morphologically distinct “stumpy” form [60].

Major parasite surface antigens (60 HC articles; Fig 4B) have received substantial attention and have emerged as major virulence factors. These antigens are often connected to the plasma membrane via glycosylphosphatidylinositol (GPI) anchors, which are themselves potent immunomodulators [61]. *Leishmania* parasites are coated with heterogeneous, GPI-anchored lipophosphoglycans (LPGs) that promote within-host parasite persistence. LPG determines sandfly vector competence, promotes adhesion, and provides protection from digestive enzymes in the sandfly midgut, while developmentally distinct forms promote survival in macrophages by suppressing signalling pathways, oxidative defence mechanisms, and phagosome–lysosome fusion [62]. In addition, LPG regulates cytokine-inducible nitric oxide synthase (iNOS), activates the inflammasome in macrophages [63], and activates natural killer T cells.

*T*. *cruzi* metacyclic parasites are coated with *trans*-sialidases that transfer sialic acid from mammalian host glycoconjugates to GPI-anchored mucin-like glycoproteins on the parasite surface and to host cell surface proteins [64]. Both the *trans*-sialidases and mucins are heterogeneous and encoded by large parasite gene families. These surface antigens are thought to serve protective and adhesive functions and to drive CD8+ T-cell responses.

African trypanosomes are always extracellular. In the mammal, they reside in the bloodstream, in adipose tissue [65], and in skin [66]. Both the insect salivary gland and mammalian stages are coated with variant surface glycoproteins (VSGs). Antigenic variation for the purpose of evading host adaptive immunity is the primary role of these GPI-anchored proteins, encoded by thousands of distinct genes. Several studies have focussed on cataloguing the VSG genes, their telomeric environment, and the diversity of VSGs expressed during immune evasion in animal models, as well as VSG recycling influenced by hydrodynamic flow [67]. Other studies describe mechanisms of VSG gene expression and allelic exclusion, the expression of a single gene from a large gene family; several proteins have been identified that are involved in this latter process [68].

Another ongoing “arms race” between African trypanosomes and their mammalian hosts, this time involving innate immune defence, emerged in relation to human-infective *T*. *b*. *rhodesiense* and *T*. *b*. *gambiense* (15 HC articles; Fig 4C). Apolipoprotein L1 (ApoL1), a component of high-density lipoprotein (HDL) particles, was found to be the human serum (*T*. *b*. *brucei*) trypanolytic factor [69], while the VSG-related proteins, *T*. *b*. *rhodesiense* SRA and *T*. *b*. *gambiense* TgsGP, were found to confer resistance to lysis. Fully susceptible *T*. *brucei* parasites capture HDL particles via their haptoglobin–haemoglobin receptor, which is also defective in *T*. *b*. *gambiense*. Further illuminating the arms race, populations of African ancestry were found to express ApoL1 isoforms with increased trypanolytic activity that may alleviate sleeping sickness symptoms [70]; a negative consequence of expressing these variants though is increased susceptibility to kidney disease [71]. This particular 2010 article, with echoes of the malaria–sickle cell anaemia story, has the highest citation rate since publication (www.scopus.com) of all trypanosomatid articles analysed here.

Extracellular vesicles (EVs, also known as exosomes, 15 HC articles; Fig 4D) are involved in cell-to-cell communication and can modulate the host immune response. EVs are produced by both insect vector and mammalian stages of all 3 trypanosomatids. Notably, a 1991 description of *T*. *cruzi* EVs preceded other HC articles by 18 years [72], revealing a more recent resurgence of interest in EVs. Proteomic analysis of *Leishmania* EVs revealed large numbers of proteins [73], while analysis of both *Leishmania* and *T*. *cruzi* EVs revealed small RNAs that can modulate gene expression in host cells. *Leishmania* EVs, which may be transmitted by sandflies along with parasites [74], are immunosuppressive through interactions with neutrophils, macrophages [73], and DCs. EVs may also facilitate transmission of the *Leishmania* RNA virus [58]. *T*. *cruzi* EVs can promote developmental transition in the triatomine bug and increase host cell susceptibility to inflammation and invasion, while host cell–derived EVs may promote invasion by *T*. *cruzi*. EVs from *T*. *brucei* promote social motility in the tsetse fly and have the capacity to transfer virulence factors to other *T*. *brucei* cells in the bloodstream as well as to cause anaemia [75].

### Vaccine research and immune responses to *Leishmania* infection

Vaccine research (48 HC articles) has focussed almost exclusively on leishmaniasis, although 2 HC articles published in 2019 focus on *T*. *cruzi* and a 2021 article provides new promise for vaccination prospects against African trypanosomes, specifically *T*. *vivax* in this case [76]. Antigens from *Leishmania* parasites [77] and antigens from sandfly saliva are both protective. Adjuvants [78] and immune responses have been investigated, primarily in mouse models but also in dogs or nonhuman primates. One particularly HC study describes a key role for CD4+ T-cell cytokine responses in vaccine-mediated protection against *Leishmania* [79], while a 2020 article provides a modern take on “leishmanization” with genome-edited and attenuated *Leishmania* parasites [80].

Several trypanosomatid research themes (CD4/8, dendritic, interleukin, nitric oxide, and Th1/2; 221 HC articles in total) relate primarily to immune responses to *Leishmania* infection in mouse models. Further immunological insights from this set and beyond those described in the “host–parasite” section above are noted here. CD8+ cytotoxic T cells can cause tissue damage or be protective but may be exhausted in visceral leishmaniasis. Regarding CD4+ T helper cells, the balance between a T helper 1 (Th1, cell-mediated) or 2 (Th2, humoral) response determines whether the infection is cleared/controlled or exacerbated, respectively [81–83]. A protective Th1 response is promoted by IFN-γ and interleukin-12 (IL-12) [84,85] and antagonised by TGF-β, while a Th2 response is promoted by IL-4 and IL-10 [86,87]. Production of nitric oxide (NO) by the type 2 inducible NO synthase limits intracellular *Leishmania* replication in macrophages [88–90], while *Leishmania* or host arginase can drive parasite replication by increasing polyamine synthesis. Induction of host arginase can also antagonise NO production, which is L-arginine dependent. Engulfment of *Leishmania* by DCs is facilitated by Fcγ and DC-SIGN receptors, allowing the DCs to present antigen and produce IL-12 and NO, thereby contributing to T cell–mediated protection. CD8+ cytotoxic T cells and NO can also be protective against *T*. *cruzi*. Research in this area reflects a sustained high level of interest in immune responses to *Leishmania* infection as well as in anti-*Leishmania* vaccine development.

### Trypanosomatid omics and genetics

Trypanosomatid genomes are typically diploid, although chromosome ploidy can vary and subtelomeric regions in particular display high rates of recombination and hemizygosity. Genetic exchange during sexual recombination in trypanosomatids (10 HC articles) appears to be restricted to the insect vector stages [91]. Other studies on DNA recombination and DNA repair have facilitated the development of new genetic tools as well as provided insights into genome organisation, virulence gene recombination, and the emergence of drug resistance.

The TriTryp genome sequences were published in 2005, yielding a set of particularly HC articles [92–95], which were followed by several further omics studies. In terms of genomics (35 HC articles; Fig 4E), studies have focussed on comparative or population genome sequencing, for additional trypanosomatid species and subspecies [96,97], and for clinical isolates [38], using short-read, long-read, or nanopore technologies. Notably, a 1999 description of the *Leishmania* chromosome 1 sequence [98] precedes other HC genomics studies by 6 years. In terms of “functional genomics,” the first-ever RNA interference library screen was conducted in *T*. *brucei*, and several subsequent articles feature genome-scale screens using this posttranscriptional gene silencing approach [99].

Trypanosomatid transcriptomics studies (34 HC articles; Fig 4F) have progressed from microarrays in 2006, to RNA-seq [100], to ribosome profiling, and, more recently, to single-cell RNA-seq [101]. Studies focus on the analysis of differentiation, life cycle stage differences, additional species and subspecies, and parasites in mammalian and vector hosts. Gene expression control (15 HC articles) is unconventional in trypanosomatids in that transcription is polycistronic, with initiation and termination sites marked by specific histone variants [102]. This places major emphasis on posttranscriptional controls mediated by RNA binding proteins. Indeed, several transcriptome studies focus on posttranscriptional controls. RNAs can also mediate control, as illustrated by a recent example of translation control by a tRNA half [103].

Trypanosomatid proteomics studies (30 HC articles; Fig 4G) [104] have progressed to deeper proteome coverage, again in distinct life cycle stages, and to organellar proteomes [105], secretomes, and phospho-proteomes. Metabolomics studies (13 HC articles; Fig 4H) have focussed on technology development [106], responses to drugs, or characterisation of metabolic pathways. Computational tools are central to these omics studies, including tools for transcriptome network analysis [107]. Indeed, the genome databases, veupathdb.org and tritrypdb.org [108], have provided critical access to omics datasets as well as access to a range of bioinformatics and in silico analysis tools.

Technologies drive discovery and the development and application of genetic tools (25 HC articles) and other methods (31 HC articles) accordingly feature prominently among HC trypanosomatid articles. Inducible gene expression has been widely used as an experimental tool in *T*. *brucei* [109], as has inducible RNA interference [110,111]. A more recent set of HC articles describes the development and application of CRISPR-Cas9 gene editing (10 HC articles) for the trypanosomatids [112,113]. A 2019 article describes the use of Cas9 to assemble >100 *Leishmania* flagellar protein mutants [114], and, as noted above, a 2020 article reports the use of Cas9 to generate attenuated *Leishmania* parasites for potential vaccination or “leishmanization” [80]. In terms of protein structure studies (24 HC articles), cryogenic electron microscopy is a technology gaining traction in trypanosomatid studies [115]. Computational tools are also central to structural studies and have been used to assess antitrypanosomal drug-binding pockets [116]. In addition, there have been substantial advances in computational approaches to 3D structure prediction, recently applied to thousands of trypanosomatid proteins (https://alphafold.ebi.ac.uk).

TriTryp genome sequencing combined with powerful genetic tools unquestionably transformed trypanosomatid research studies, in the areas of molecular and cell biology in particular, and provided key insights into eukaryotic evolution (21 HC articles). Other exemplar studies that illustrate the impact of omics include studies on the trypanosomatid flagellum (14 HC articles) and cell motility [105,114] and the discovery of unconventional kinetochores [117], the macromolecular complexes that direct chromosome segregation, that likely diverged from conventional kinetochores at the root of the eukaryotic tree of life. Continuing the unconventional theme is polycistronic transcription [118] as described above, and compartmentalisation of glycolysis (11 HC articles) within glycosomes [119], considered over many years to represent a promising target for chemotherapy. The kinetoplast, a complex mitochondrial genome comprising a network of interlocked maxicircles and minicircles, after which the Kinetoplastida group of flagellated protists are named, is another unconventional feature and an established drug target. Several studies focus on mechanisms of kinetoplast DNA (kDNA) replication and segregation. In addition, the unique, high-copy number kDNA sequences have been exploited as targets for diagnostic tests, while the kDNA and the flagellum have long served as prominent features under the microscope that have greatly facilitated studies on cell cycle progression.

Trypanosomatids have additionally served as “model eukaryotes” in relation to conserved organelles, molecules, machineries, and processes that were first discovered in these parasites. Examples include acidocalcisomes [59], thought to be involved in osmoregulation; the GPI membrane anchors [120] described above; mRNA editing (11 HC articles), which is extensive within transcripts derived from the mitochondrial kDNA genome [121]; *trans*-splicing [122], which occurs transcriptome-wide in conjunction with polycistronic transcription in trypanosomatids; and epigenetic control by oxidation of 5-methyl groups on DNA bases [123] catalysed by enzymes with homologues in mammals. Studies on trypanosome flagellar defects also provide models for understanding human ciliopathies with analogous underlying defects [124].

### Research trends and emerging topics

**Fig 5:**
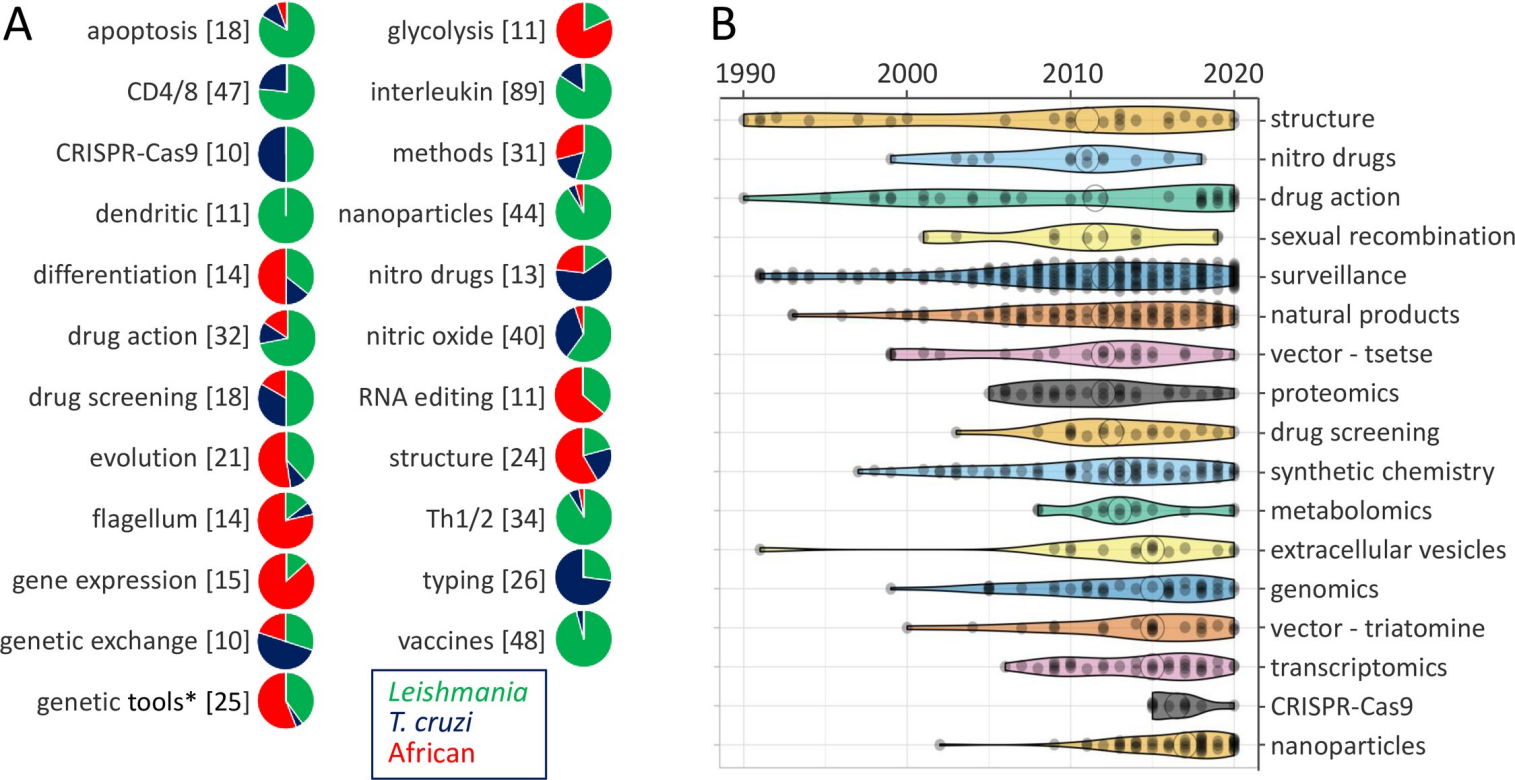
Research trends and emerging topics. (**A**) Trypanosomatid research topics represented in the set of HC articles and not shown in Figs 3 and 4. The pie charts indicate the distribution of outputs for each topic among the trypanosomatids. Number or articles in each set is indicated in square brackets. * excluding “CRISPR-Cas9” articles. (**B**) The violin plot shows those topics for which the HC articles have a median publication date later than 2010. Topics are ranked by median value as indicated by the open circles. HC, highly cited.

The trypanosomatid research community has strengthened key areas and has continued to deliver new research topics and technologies. Fig 5A lists all of those areas and topics detailed above that do not appear in Figs 3 and 4 and the distribution of outputs for each topic among the trypanosomatids, while Fig 5B shows all topics for which the HC articles have a median publication date later than 2010. Some of these latter topics and research areas have a long history and are experiencing an increasing level of interest more recently, such as protein structure determination, drug mechanism-of-action studies, and surveillance studies. Natural product studies, drug screening, and nanoparticle formulation studies all register many recent and HC articles, and there has also been a growing interest in the insect vectors. As noted above, omics studies followed publication of the TriTryp genome sequences in 2005, and the development and application of CRISPR-Cas9 genome editing tools emerged even more recently.

## Concluding remarks

**Fig 6:**
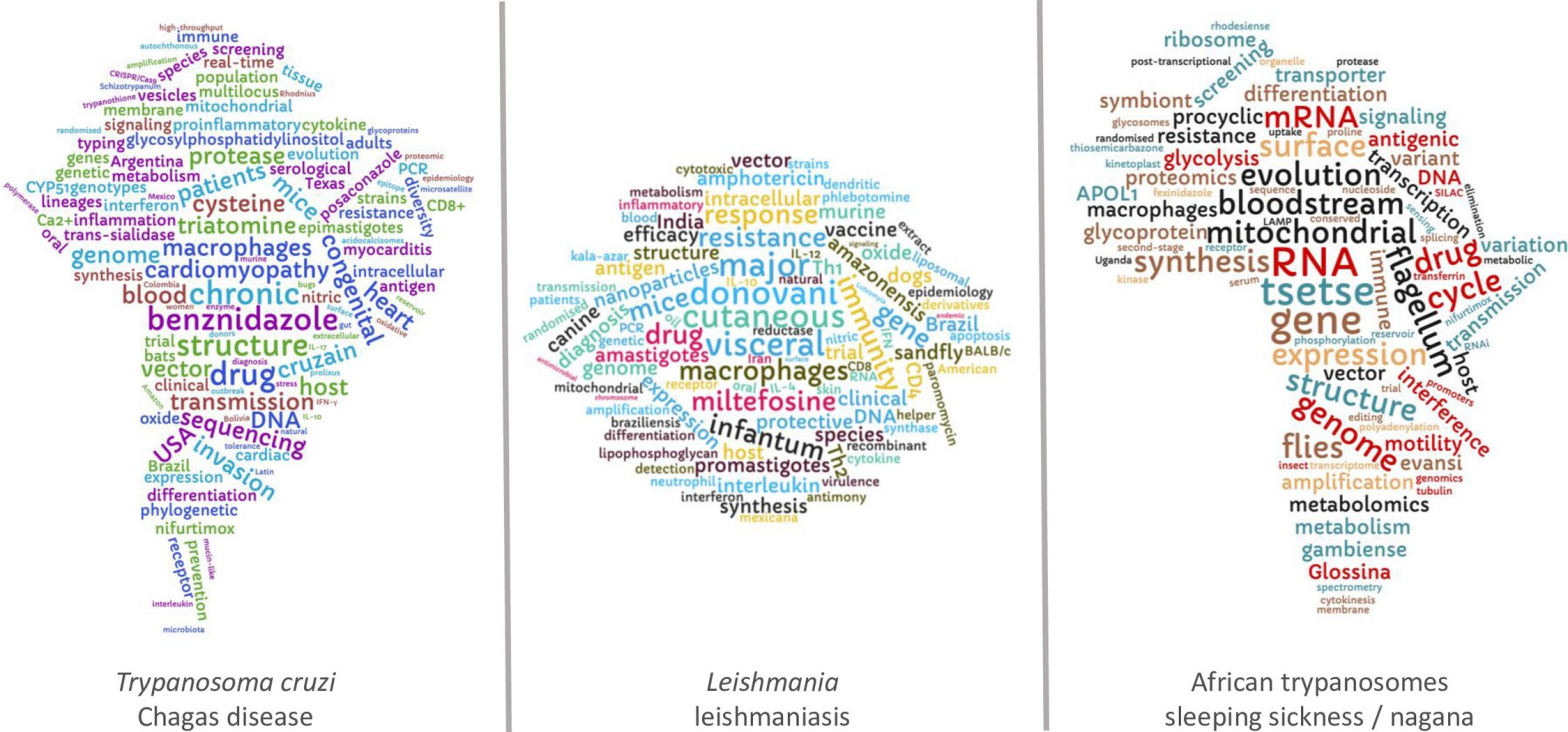
Word clouds. Word clouds for each trypanosomatid group derived from the titles of HC articles from 1990–2020; data from Scopus. Trypanosomatid and disease-associated terms used to search Scopus were removed. HC, highly cited.

The goal here has been to include a broad range of topics and to provide a representative and inclusive profile of trypanosomatid research. The word clouds (Fig 6), derived from the set of 1,786 HC article titles, capture even more topics. Use of citation rate is an imperfect method for capturing a fully representative profile of trypanosomatid research, however. Some articles have a broader scope that extends beyond trypanosomatids, for example. These include assessments of compound collections, either natural or synthetic, which often incorporate assays with other pathogens and/or cancer cell lines, and studies that primarily focus on immune cell function, inflammation, or the response to pathogen challenge. There are also certainly thousands of excellent research articles not captured in the set analysed here. Nevertheless, a focus on primary research articles and a quantitative approach using citation metrics has clearly captured a broad range of topics on both the parasites and the diseases they cause. The exemplar studies cited additionally capture both the discovery science and the translational research.

The trypanosomatid research community has made great strides in the past and continues to do so. Intimately linked to these advances are vector control efforts, improved diagnostics, and increasingly populated clinical development pipelines (https://dndi.org/research-development/portfolio/). Indeed, although challenges remain, control and elimination prospects now look increasingly encouraging for these NTDs [1–3]. The outputs and successes detailed above reflect a vibrant and dynamic trypanosomatid research community that has strengthened key areas and has continued to deliver new research topics, trends that are set to continue.

**Key Learning Points**

- Trypanosomatid research is vibrant and diverse globally in both endemic and nonendemic countries.
- Translation and drug discovery are having a substantial impact, facilitated by public–private and industry partnerships.
- Many new discoveries and new research areas have emerged, often facilitated by technological advances.

**Top Five Papers**

1. Belkaid Y, Piccirillo CA, Mendez S, Shevach EM, Sacks DL. CD4+CD25+ regulatory T cells control *Leishmania major* persistence and immunity. Nature. 2002;420:502–7.
2. Genovese G, Friedman DJ, Ross MD, Lecordier L, Uzureau P, Freedman BI, et al. Association of trypanolytic ApoL1 variants with kidney disease in African Americans. Science. 2010;329:841–5.
3. Aslett M, Aurrecoechea C, Berriman M, Brestelli J, Brunk BP, Carrington M, et al. TriTrypDB: a functional genomic resource for the Trypanosomatidae. Nucleic Acids Res. 2010;38:D457–62.
4. Khare S, Nagle AS, Biggart A, Lai YH, Liang F, Davis LC, et al. Proteasome inhibition for treatment of leishmaniasis, Chagas disease and sleeping sickness. Nature. 2016;537:229–33.
5. Mesu V, Kalonji WM, Bardonneau C, Mordt OV, Blesson S, Simon F, et al. Oral fexinidazole for late-stage African *Trypanosoma brucei gambiense* trypanosomiasis: a pivotal multicentre, randomised, non-inferiority trial. Lancet. 2018;391:144–54.
